# Fertility is below replacement in Harar Health and Demographic Surveillance System (Harar HDSS), Harar town, Eastern Ethiopia

**DOI:** 10.1186/s40738-016-0023-8

**Published:** 2016-06-09

**Authors:** Nega Assefa, Agumasie Semahegn

**Affiliations:** College of Health and Medical Science, Haramaya University, P.O. Box 1494, Harar, Ethiopia

**Keywords:** Fertility, Fertility rate, Women, Health and Demographic Surveillance System, Harar, Ethiopia

## Abstract

**Background:**

Population growth is determined by fertility, mortality and migration rates. Fertility is the prime determinant of population growth, which is highly associated with family planning, literacy, urbanization, and expansion of health system. In many part of Africa, its level is more than twice the replacement level. In Ethiopia, a significant decline in fertility mainly in the urban setting has been reported over the past decade, yet there is a paucity of information on the level of the decline. Therefore, this analysis aims to assess the level of fertility in Harar Health and Demographic Surveillance System (Harar HDSS) Eastern Ethiopia.

**Methods:**

Harar HDSS is an urban HDSS located in the city of Harar, eastern Ethiopia. It was established in 2011. All the population under surveillance are followed regularly and updated every six month for any change in the population demographic characteristics. Data were collected on a face-to-face interview to record demographic and socio-economic characteristics. Data were entered into customized HRS-2 software used for capturing longitudinal data and exported to computational software for analysis. For this analysis fertility data of the year 2013 were used. Fertility levels were analyzed using descriptive statistics.

**Results:**

The total population of Harar HDSS in 2013 was 30,055. Of these, 15,701 (52.2 %) were females and 14, 354 (47.8 %) were males. The crude birth rate and general fertility rate for the year 2013 were 20.3 and 64 births per 1000, respectively. In 2013, the Total Fertility Rate (TFR) was 1.9 births per women of reproductive age. The 25 to 29 age group has the highest age-specific fertility rates (128.1 births per 1000 women), followed by the 20 to 24 year old women (89.3 births per 1000 women).

**Conclusion:**

Total fertility rate was relatively low. However, there were a significant number of births among adolescent women. Improving and sustaining access for reproductive health care for young women is highly recommended.

## Background

Africa began experiencing fertility decline associated with demographic transition in the 1980’s, lagging behind the other developing regions of Asia and Latin America (which declined in 1960–1970s) and the developed world of North America and Europe (which declined prior to the 1970s) [1–3]. The demographic transition refers to the shift in a population’s demographics from high mortality and high fertility to low mortality and low fertility levels. In the accepted transition model, fertility decline lags behind mortality decline causing periods of rapid population growth, as is the case in Africa currently [3–5]. Ethiopia is no exception to this trend. This time the country is assumed to be in the second stage of the demographic transition, defined by falling death rates with mostly unchanged birth rates, resulting in sustained, rapid population growth [6]. The population, when last recorded in the 2007 census, has been growing at a rate of 2.6 % per year over the past eighteen years [7]. This is especially true for 84.9 % [7] of the population that resides outside urban areas. Data from the nearby rural Kersa Health and Demographic Surveillance (Kersa HDSS) showed that the population is increasing at a rate of 2.5 % per year since surveillance began in 2007 [8].

Population growth is determined by fertility, mortality and migration rates, while fertility being the prime determinant of population growth [4]. Not only do fertility rates directly add more people to the population, but past high fertility rates mean that cohorts of individuals in their reproductive years are larger and thus will produce more children [3, 6]. Sub-Saharan Africa, being at the beginning of the demographic and fertility transition, has had relatively slow fertility decline in the last couple of decades of the 20^th^ century, when compared to other regions at the same stage of the transition [1]. Some even believe that decline has stalled in recent years [9]. Since Africa began its demographic transition, continued fertility decline from a high of 6.5 children per woman in 1985 [9] to 5.1 children per women in 2005–2010 has been documented [1]. Even with this decline, fertility levels still remain high throughout much of the continent when compared to the rest of the world at both their current stage of fertility transition and at the beginning of the transition [1]. The continent has the highest fertility rates in the world [10]. Most of the fertility decline in Africa has been concentrated in Northern Africa and urban areas [2]. Sub-Saharan Africa, still boasts fertility level about twice the replacement fertility and this has been a huge driving force in its continued rapid population growth [1].

In Ethiopia, the total fertility rate in 2011 was approximately 4.8 children per women, but ranges from 2.6 children per women in urban areas to 5.5 children per women in rural areas [7]. While fertility rates in the large urban areas such as Addis Ababa, have been recorded as declining to below replacement levels [11], the rest of the country still has a high Total Fertility Rates. For example in Somali and Oromia regions the TFR is 7.1 [7] and 5.1 children per women, respectively. These are the two highest fertility rates of all of the regions in Ethiopia [7]. Results from the 2011 E DHS showed that there is a decline in the fertility rate from the preceding reports. The overall, fertility rate in Ethiopia dropped from 5.5 children per woman in 2000 to 4.8 children per woman in 2011 [7]. Much of this decline had occurred between 2005 and 2011 [7]. The EDHS 2011 report indicated that the TFR for Harari region, where the current study is conducted, was 3.4.

In the scenarios above, it is understood that fertility is declining and in some places it is below replacement. The purpose of this paper is to understand if the same conditions exist in other urban centers in Ethiopia outside Addis Ababa. Harar HDSS provide a robust platform for observing the changes in fertility over time through constant longitudinal surveillance. Therefore, the aim of this analysis was to determine the level of fertility in the Harar Health and Demographic Surveillance System (Harar HDSS) in 2013, to better understand how fertility has impacted population growth in the area during this period.

## Methods

### Study setting and period

**Fig. 1:**
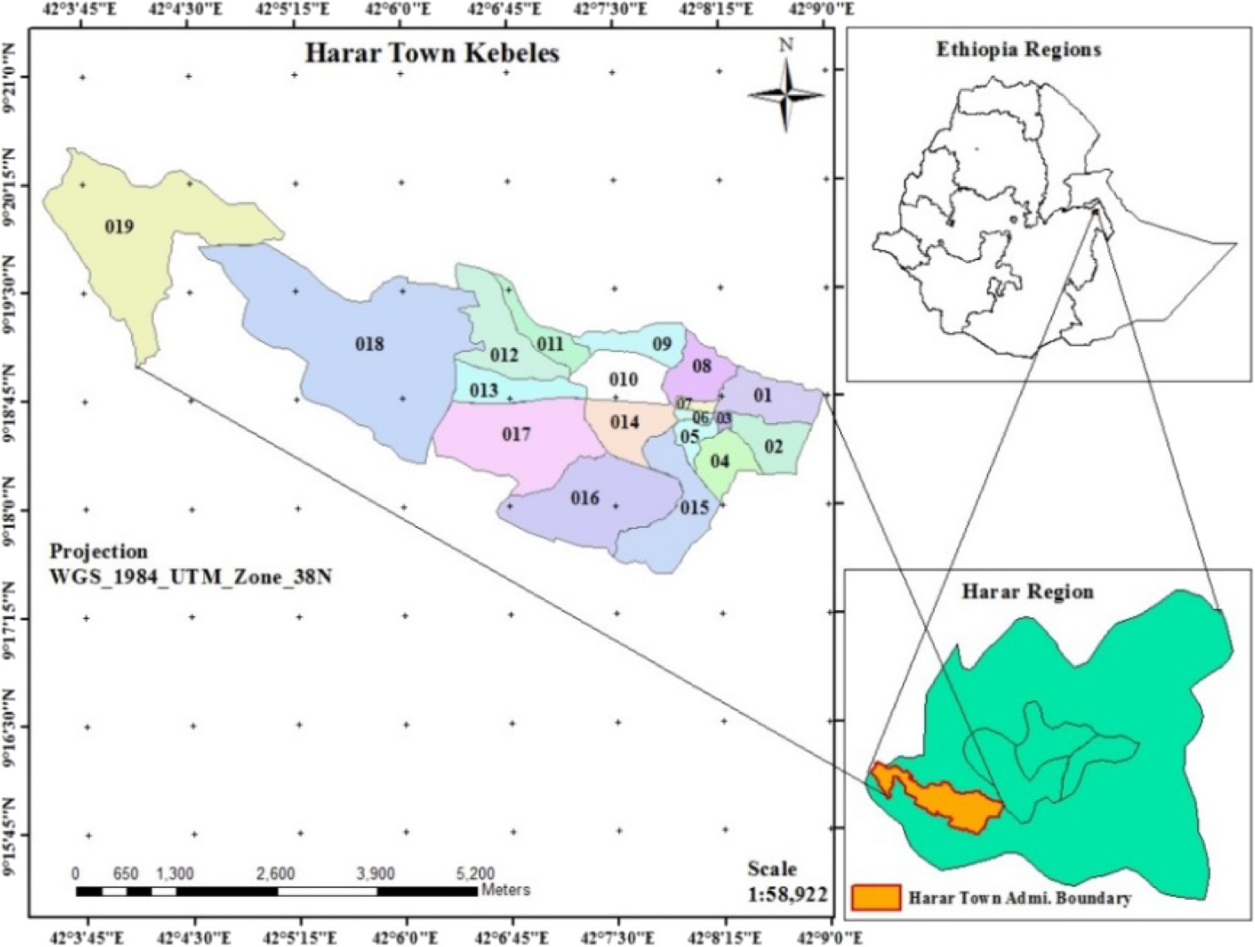
Map of Harar, East Ethiopia

The study was conducted in Harar city, urban HDSS [8, 12] which is located in Harari People National Regional State, Ethiopia. It is located in the Eastern part of Ethiopia, 510 km from Addis Ababa. With a total area of 343.2 square kilometers (Urban = 19.5Sq Km and Rural = 323.7 Sq Km), it is the smallest region in the Federal State of Ethiopia. The region is bordered with some districts of the Eastern Zone of Oromia Regional, Kombolcha and Jarso districts in Northern, Gursum and Babile districts in the East, Haramaya in the West and Fedis in the South (Fig. 1).

According to the current administrative structure, Harari Region is divided in to six urban and three rural administrative districts. These administrative districts are further divided in to 19 Kebeles (in the urban area) and 17 Kebeles (in rural areas) (Kebele is the smallest administrative unit in Ethiopia with an average population of 5000). Harar city is one of the major urban areas of the region. According to the 2007 national population census the total population of the region was 183,344 of which 99,321 (54.2 %) were urban and 84,023 (45.8 %) were rural residents. About 62 % of the population resides in the urban area including Harar. The population growth rate of the region for the year 2007–2010 for urban and rural areas was estimated to be 2.0 and 3.3 %, respectively. The average gross population density of the region is estimated to be 552 persons per KM^2^, while the average estimated gross density is 6012.4 per KM^2^ and 222.5 per KM^2^ for urban and rural areas, respectively.

### Study design and sampling procedure

Longitudinal community based follow-up of the population is the main method of HDSS. The survey was conducted twice a year to get completed data. In September 2011 a baseline census that covered the selected six Kebeles was conducted and follow up of the demographic and health related events started soon after the census. Before conducting the baseline census, the task of the enumerators was to identify the boundaries of the selected Kebeles. They were provided with paper and pencils to hand draw the route or order of the households they visited. Drawing boundaries and marking key landmarks or features of their enumeration area greatly helped them in maintaining the order of future visits for the baseline census and in the subsequent updates. After the map was created and households were identified, the residential houses were numbered. Each enumerator was provided a hand-held GPS and the households were then geo-referenced. The first census was conducted in September 2011 and subsequent round updates are being done to date. For this analysis, data from the two rounds of 2013 update was extracted from the main database.

### Data collection procedure

The population has been followed twice a year (every 6 months) since surveillance began in 2011. Twelve data collectors who were secondary school graduates (grade 10^th^ and above) with some experience in field survey and 3 diploma nurse supervisors were recruited to conduct the census and monitor/supervise the data collection process, respectively. The data collectors were purposely assigned to their residence Kebeles to ease the data collection and event identification process. The boundaries of each of the Kebeles were identified and mapped and all houses were numbered. Data were collected using structured questionnaires adapted from other HDSS sites. In every round, competent household members was contacted to respond to questions. The data collection included socio-economic characteristics, observation and measurement of physical structures and facilities of houses in addition to the vital event questions. The questionnaires were prepared in English and later translated to Afan Oromo and Amharic language and then back to English by independent translators to check consistency. The data collectors and supervisors were trained for one week and the data collection instruments were pre-tested. All interviews are carried out in the local languages, Amharic or Afan- Oromo, but saved in English in the database. Vital events were recorded during every visit. Additionally, at each visit pregnancy is asked and registered. If a birth had occurred, a ‘Birth Registration’ form is completed by the data collector.

### Data processing and statistical analysis

Prior to data entry and analysis, each data collector submitted completed questionnaires to their supervisor daily to be checked for completeness and consistency. Incomplete questionnaires were returned to data collectors immediately for correction. The collected data were double entered into computer by two data clerks and validated; and the inconsistencies were traced and corrected. The data in the year 2013 was exported from HRS-2 to statistical packages for analysis. Descriptive statistics were carried out after cleaning the inconsistencies in the data to determine level of fertility using Microsoft Excel ©. The total birth events were measured between the end of this year as well as information about the age and marital status of the mother. The General Fertility Rate (GFR), Crude Birth Rate (CBR) and Total Fertility Rate (TFR) were calculated. GFR is total births divided by the number of women of reproductive age; CBR is the total births divided by the total midyear population, and TFR is the total number of births women in that community give to, which is calculated indirectly from the summation of the Age Specific Fertility Rate (ASFR) and multiplied by five. The constant in all the cases was 1000 [1]. ASFR is like GFR, but calculated for specific age groups divided in five year intervals.

The denominators used in all these rates represent the mid-year population estimates for the surveillance year 2013.

## Results

In 2013 the total population of Harar HDSS was 30,055. Of these, 15, 701 (52.2 %) were females and 14, 354 (47.8 %) were males. The sex ratio was 91.4 % (9:10). A total of 610 births were observed during the year. Of these, 291 (47.7 %) were female and 319 (52.3 %) were male births. Two births were in women under 15 years old (Table 1). The crude birth rate was 20.3 births per mid year population in 2013. The total fertility rate was 1.9 births per women (15–49 years of age) in 2013. The general fertility Rate (GFR) was 64 births per 1000 women aged 15 to 49 years in 2013 (Table 2).

**Table 1:** Birth distribution by maternal age in Harar Health and Demographic Surveillance System (Harar HDSS), 2013

Age	Number of women	Number of births
15–19	1700	37
20–24	1780	159
25–29	1780	228
30–34	1663	136
35–39	1120	33
40–44	981	11
45–49	517	4
Total	9541	608

**Table 2:** Fertility Rates in Harar HDSS, Kersa DSS, Oromia, and Ethiopia

	Harar HDSS	Kersa HDSS	Oromia*	Ethiopia*
TFR	1.9	5.3	4.85	4.8
GFR	64	166.8	153.30	128.80
CBR	20.3	37.2	33.90	30.10

*SOURCE: 2007 Census (Central Statistical Agency [Ethiopia], 2010)

**Fig. 2:**
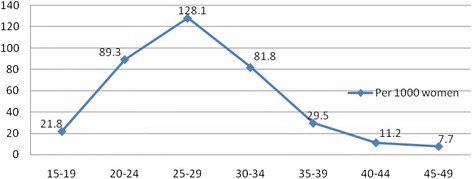
Age Specific Fertility Rate among women at Harar HDSS, 2013

The age specific fertility rate showed that there was variation in fertility rates across women’s ages. Two births observed among early adolescent women (less than 15 years old). The late adolescent fertility (15–19 years old) was 21.8 births per 1000 women. The 25 to 29 age group had the highest age-specific fertility rates (128.1 births per 1000 women) in 2013, followed by the 20 to 24 age group (89.3 births per 1000 women). Rates for all other age groups were relatively low, with the lowest rate recorded for the older age group (45–49), 7.7 births per 1000 women (Fig. 2).

**Fig. 3:**
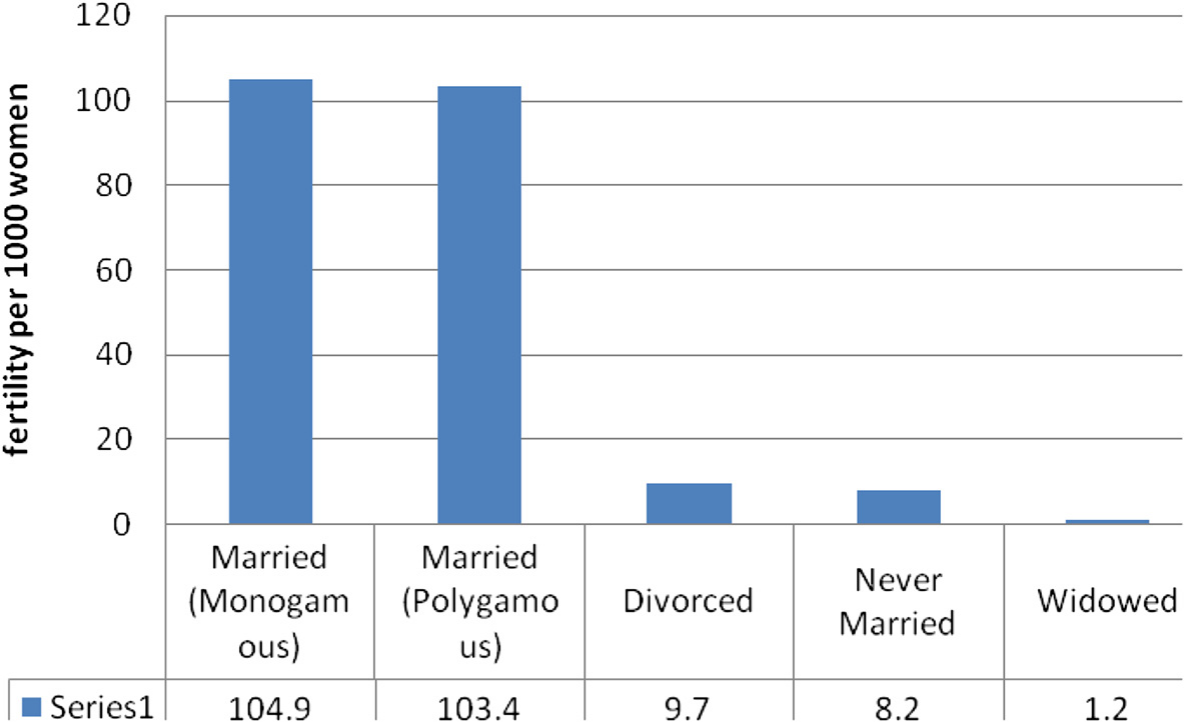
Feritility status of women by marital status at Harar HDSS, 2013

Fertility by marital status showed that, the highest fertility rate is recorded for married women (monogamous) that were 104.9 births per 1000 women, followed by married (polygamous) 103.4 births per 1000 women. The lowest fertility rate is recorded for widowed women, which was 1.2 births per 1000 women (Fig. 3).

**Fig. 4:**
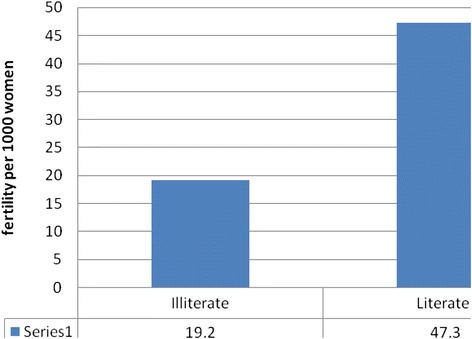
Educational status of women by fertility status Harar DSS, 2013

There were significant differences between literate and illiterate women. Fertility was quite high among literate women at 47.3 birth per 1000 women (Fig. 4). The literate women had more births compared to the illiterate; as the site is urban, more literate women were expected.

## Discussion

The Total Fertility Rate for Harar H DSS in 2013 was much lower than expected considering the national average and Oromia region where Kersa HDSS is located. Two offspring from couples are expected to sustain the population assuming no death. In reality, there is a risk of death; hence to compensate for the loss, a bit more than a fertility rate of 2 per women during her reproductive life is needed to sustain a population [13].

The community based longitudinal study design and regular follow up of vital events are strengths of this study. However, it is difficult to compare the fertility rates between literate and illiterate women because most urban residents have some exposure to formal education, which means there are few illiterate women to compare with. Harar town is one of the oldest cities in Ethiopia and modern education is more established than in other parts of the country.

In Table 2, fertility characteristics of different parts of the country is compared with Harar HDSS. Harar HDSS is located in an urban setting, hence it has the lowest fertility rate compared to Kersa HDSS and other parts of Ethiopia. In the urban setting, where access for education and health service including abortion and family planning service is high, fertility declines [8, 14, 15].

Ethiopia, in general,  is in the second stage of demographic transition. During this demographic transition phase fertility is high and mortality is low. With the advancement of urbanization and better access to improved health service, it is possible for urban centers to move to stage three of the demographic transition where fertility is declining and mortality is low. The latter phenomenon is occurring in Harar. This transition is highly associated with increased education, better access to family planning and abortion services [7, 8, 16]. As this analysis is based on data from an urban HDSS, we expected this phenomena. A limited time period for the analysis gives a limited understanding of the case in time. Better understandings is established if data for several years were evaluated, which is premature for Harar HDSS. Hence, it is recommended to extended surveillance of the area over time to illuminate trends in fertility.

Even though the fertility rates is lower than expected, important characteristics of fertility were comparable to the country’s trends of fertility reduction [15, 16]. The urban nature of Harar and the possibility of access to health systems and modern education, may explain reduction in fertility [17, 18]. Majority of women in Harar H DSS who gave birth were married and did so at fairly young ages. In Ethiopia, marriage, age at first birth and age at first sex are closely related, especially for women [7]. Women married at fairly young ages, especially in rural areas, and gave birth soon after. The average age of first marriage in Ethiopia was 17.1 years for women aged 20 to 49 years and 17.4 years in the Oromia region in 2011 [7]. Marriage is almost universal practice in the country with 57.7 % of women being married by age 18 and 91.4 % by age 25 in 2011 [7]. Conversely, the median age at first sexual intercourse was 17.1 years for women aged 20 to 49 years in 2011, which aligned with the age of first marriage [7]. This suggests that sex occurs primarily within marriage. The median age of first birth occurred at 19.6 years in 2011, about 2 years after marriage [7]. This trend is also Kersa HDSS. In this analysis, women gave birth young and within marital union.

Though several factors impact fertility, family planning and abortion are key determinants in shaping the fertility level in the urban setting. This days, family planning is in the reach of every household through house to house visits by the health extension workers and easily accessible mother and children clinics. Hence access to family planning service is nearly universal, yet the actual use of family planning is fairly low. The health extension program is designed to provide basic health promotion activities. The services rendered by the health extension program includes sixteen health promotion packages including family planning, antenatal, delivery and postnatal care [19, 20]. This program has been exemplary in addressing the health needs of populations in rural and urban slum areas in Ethiopia [21].

Obtaining abortion service is as easy as obtaining family planning services in Harar after reform in the provision of abortion service in Ethiopia. Every health center is equipped and staffed with manual vacuum aspiration (MVA) and trained health professionals. Similarly every hospital in town is able to give the service. As indicated in many studies, the easy access to family planning service and abortion facilities could have positively impacted the reduction in fertility rates.

Other factors such as age of first marriage/first sex, prevalence of sexually transmitted diseases and their effect on sterility, value of children, economic development and women’s status are important factors impacting the overall fertility rate [3, 10, 13, 22, 23]. Investigating how these various factors affect fertility preferences and trends in the area is beyond the scope of this analysis but would help in shedding light on future changes in fertility in the area.

## Conclusion

A one year total fertility rate in the Harar HDSS was relatively lower than the replacement level which is 2. The highest age specific fertility rate observed among women whose age in 20s. Married (monogamous) women have highest fertility followed closely by married polygamous women. The fertility rate in Harar HDSS is lower than Harari Region and Ethiopia’s overall fertility rate.
